# The association between parental history of diagnosed mood/anxiety disorders and psychiatric symptoms and disorders in young adult offspring

**DOI:** 10.1186/1471-244X-12-188

**Published:** 2012-11-05

**Authors:** Nancy CP Low, Erika Dugas, Evelyn Constantin, Igor Karp, Daniel Rodriguez, Jennifer O’Loughlin

**Affiliations:** 1 Department of Psychiatry, McGill University, 1033 Pine Avenue West, Montréal, QC, H3A 1A1, Canada; 2 Centre de Recherche de Centre Hospitalier de l’Université de Montréal, 3875 Saint Urbain, Montréal, QC, H2W 1V1, Canada; 3 Department of Pediatrics, McGill University, Montreal Children’s Hospital, 2300 Tupper Street, Room C-538E, Montréal, QC, H3H 1P3, Canada; 4 Department of Social and Preventive Medicine, University of Montréal, 7171 Parc Avenue, Montréal, QC, Canada; 5 Centers for Behavioral and Preventive Medicine, Department of Psychiatry and Human Behavior, Alpert Medical School, Brown University, The Miriam Hospital, Coro West, Suite 309, 164 Summit Avenue, Providence, RI, 02906, USA; 6 Institut national de santé publique du Québec, 190 Crémazie Blvd. East, Montréal, QC, H2P 1E2, Canada

**Keywords:** Familial risk, Parental history, Offspring, Psychiatric, Mood, Anxiety, Panic

## Abstract

**Background:**

Parental history of mood or anxiety disorders is one of the strongest and most consistent risk factors for the development of these disorders in offspring. Gaps remain however in our knowledge of whether maternal or paternal disorders are more strongly associated with offspring disorders, and whether the association exists in non-clinical samples. This study uses a large population-based sample to test if maternal or paternal history of mood and/or anxiety disorders increases the risk of mood and/or anxiety disorders, or symptoms of specific anxiety disorders, in offspring.

**Methods:**

Data were drawn from the Nicotine Dependence in Teens Study, a prospective cohort investigation of 1293 grade 7 students. Data on mental health outcomes were collected in mailed self-report questionnaires when participants were aged 20.4 (0.7) years on average. Parental data were collected in mailed self-report questionnaires. This current analysis pertains to 564 participants with maternal and/or paternal data. The association between maternal and paternal history and each of diagnosed anxiety disorder, diagnosed mood disorder, and symptoms of specific anxiety disorders in offspring was studied in multivariate logistic regression.

**Results:**

A higher proportion of mothers than fathers had a diagnosed mood/anxiety disorder (23% versus 12%). Similarly, 14% of female offspring had a diagnosed mood/anxiety disorder, compared to 6% of male offspring. The adjusted odds ratio (95% confidence interval) for maternal history was 2.2 (1.1, 4.5) for diagnosed mood disorders, 4.0 (2.1, 7.8) for diagnosed anxiety disorders, and 2.2 (1.2, 4.0) for social phobia symptoms. Paternal history was not associated with any of the mental health outcomes in offspring.

**Conclusion:**

Maternal, but not paternal mood/anxiety disorders were associated with diagnosed psychiatric disorders, as well as symptoms of specific anxiety disorders, in offspring. Efforts to detect mood and anxiety disorders in offspring with a maternal history should be encouraged.

## Background

Mood and anxiety disorders are chronic conditions that affect up to 15-20% of adolescents and young adults [1-3]. These disorders are associated with academic failure [4-6], difficulties finding and sustaining employment [7,8], problems in relationships with family, friends and romantic partners [6,9-12], substance misuse [13-15], and suicide [16-18]. Although onset of symptoms of mood and anxiety disorders often occurs during childhood or adolescence [2,19,20] diagnosis and treatment may not occur until up to 14 years later for mood disorders, and 30 years later for anxiety disorders [21]. The years between first symptoms and diagnosis represent a critical period for preventive intervention and treatment which could reduce the future burden of mood and anxiety disorders.

Parental history of mood and anxiety disorders is one of the strongest and most consistent risk factors for the development of these disorders in offspring [22-24]. Based on findings from clinical samples (i.e., in which either parents or offspring are recruited in treatment settings), the increased risk to offspring with a parental history relative to offspring with no parental history, ranges from 2 to 4-fold for depression [25-34] and from 2 to 7-fold for anxiety [29,31,32,35-37] including panic [34,38], social phobia [38] and generalized anxiety disorders [34,38,39]. The increase in risk is greater for maternal versus paternal history [27-30] although not all studies report differences [26,28]. In addition to inconsistent findings across studies, most studies that use clinical samples are limited by small sample sizes, examination of one parent only (predominantly the mother), or assessment of offspring who may not yet be old enough to manifest illness.

In contrast to studies of clinical samples, reports based on population-based samples in which self-report or direct assessments of psychiatric disorders are collected from both parents and offspring are rare. Studies that do exist [40-44] report a range in risk from no elevated risk to an increase in risk of 1.5 to 2.5-fold. Again, inconsistencies are reported in terms of maternal versus paternal history. Two studies suggest maternal but not paternal associations, [40,42] and two suggest both [41,44]. To date, no population-based studies describe the risk of parent self-reported psychiatric history on specific offspring anxiety disorders (i.e. panic, social phobia and generalized anxiety disorders). Thus, it remains unclear whether the association between parental history and offspring psychiatric disorders exists in population-based samples of youth.

The objective of this study is to test if maternal or paternal history of diagnosed mood or anxiety disorders is associated with the risk of mood or anxiety disorders, or with symptoms of specific anxiety disorders in a large population-based cohort.

## Methods

Data were drawn from the Nicotine Dependence in Teens (NDIT) Study, a prospective cohort investigation of 1293 students recruited in 1999–2000 from all grade 7 classes in a convenience sample of 10 secondary schools in Montreal, Canada [45]. Self-report questionnaires were administered in classrooms every 3 months during secondary school from grade 7–11, for a total of 20 survey cycles during the five years of secondary school. In 2007–8, when participants were aged 20.4 (0.7) years on average, data were collected in mailed self-report questionnaires from a total of 880 participants (68% of 1293 participants) in survey cycle 21. In addition, in 2009–10, data were collected from parents in three mailed self-report questionnaires including a questionnaire for mothers (n=597; 97.7% biological mothers), a questionnaire for fathers (n=478; 97.1% biological fathers), and a questionnaire which was completed by either parent that collected data about the NDIT participant (n =647).

This current analysis pertains to NDIT participants (herein referred to as offspring) who completed survey cycle 21 and whose parents completed the self-report parental questionnaires. Two datasets were constructed, one that consisted of offspring and maternal self-report data, and the second that consisted of offspring and paternal self-report data (referred to herein as the “mother database” and “father database”, respectively). The mother dataset consisted of 564 (64% of 880 eligible offspring) participants with complete data, and the father dataset consisted of 454 (52% of 880 eligible offspring) participants with complete data. All offspring and parents provided informed consent. The study received ethics approval from the Montreal Department of Public Health Ethics Review Committee, the McGill University Faculty of Medicine Institutional Review Board, and the Ethics Research Committee of the Centre de Recherche du Centre Hospitalier de l’Université de Montréal.

### Study variables

Socio-demographic data pertaining to offspring included age, sex, language spoken most often at home (French, other), mother’s level of education (attended university, did not attend university), annual household income (<$30,000, $30,000-$99,999, ≥$100,000), and born in Canada (yes, no).

Data on diagnosed mental health disorders (yes, no) among mothers and fathers were collected in the parent questionnaires by: “Have you ever been diagnosed by a health professional with any of the following…”: (i) anxiety disorder (phobia, obsessive-compulsive disorder, panic attacks, generalized anxiety disorder (GAD)); (ii) depression; (iii) bipolar disorder; and (iv) postpartum depression (for mothers only). Data on age at diagnosis were collected if the parent endorsed being diagnosed by a health professional. For multivariable analyses, anxiety disorders, depression, bipolar disorder and postpartum depression were grouped into a single variable termed “anxiety or mood disorder”.

Diagnosed mental health disorders (yes, no) among offspring were measured in the mailed self-report questionnaire in survey cycle 21 by: “Has a health professional ever diagnosed you with the following…”: (i) mood disorder (depression, bipolar disorder); (ii) anxiety disorder (phobia, fear of social situations, obsessive-compulsive disorder, panic disorder, GAD). Data on age at diagnosis were collected if the offspring endorsed being diagnosed by a health professional.

Lifetime anxiety symptoms of panic disorder, GAD, and social phobia among offspring were assessed in survey cycle 21 using the Composite International Diagnostic Interview (CIDI) screening questions [46]. Symptoms of panic disorder (yes, no) were assessed by “Have you ever had any of the following”: (i) attack of fear or panic when all of sudden you felt very frightened, anxious or uneasy; and (ii) attack when all of sudden, you became dizzy, very uncomfortable, short of breath, dizzy, nauseous, your heart pounded, or you thought that you might lose control, die or go crazy. Symptoms of GAD (yes, no) were assessed by “Have you ever had any of the following”: (i) a time when you were a “worrier” (when you worried a lot more about things than other people with the same problem); (ii) a period lasting 6 month or longer when you were anxious or worried most days?; (iii) a time when you were much more nervous or anxious than most other people with the same problems. Symptoms of social phobia (yes, no) were assessed by “Have you ever had any of the following”: (i) a time when you felt very afraid or really shy meeting new people, going to parties, going on a date; and (ii) a time when you felt very afraid or uncomfortable when you had to do something in front of a group of people (giving a speech, speaking in class). Data on age at onset of symptom(s) were collected if the offspring endorsed ever having the symptom(s). For each anxiety subtype, offspring who responded “yes” to at least one symptom within each anxiety subtype were categorized as screening positively for that anxiety subtype. The sensitivity and specificity of the CIDI anxiety disorder screening questions compared to full diagnoses generated by the administration of the complete CIDI instrument range from 89-95% and 51-98%, respectively [47].

### Data analysis

Differences in socio-demographic characteristics between participants retained and not retained for analysis were tested in two-sample t-tests for continuous variables, and in chi-square tests for binary variables.

Because early analyses suggested that there was no statistically significant interaction between offspring sex and either maternal or paternal history of mood and/or anxiety disorders, all multivariate analyses were pooled by sex. The independent association between each of maternal history and paternal history and each of the five (binary) outcome variables (i.e., offspring diagnosed anxiety disorder, offspring diagnosed mood disorder, and offspring symptoms of panic disorder, social phobia, and GAD) was assessed in separate multivariate logistic regression models controlling for maternal education and offspring sex (i.e., the only socio-demographic variables associated with the mental health outcomes at p≤0.25 in univariate analyses (data not shown)). In secondary analyses, parental history of depression, bipolar and anxiety disorders were studied as separate exposures (Additional file 1: Table S1). In the paternal history models, we also tested for an interaction between fathers living with offspring (duration in years) and paternal history. All analyses were conducted using SAS software, Version 9.2.

## Results

While several socio-demographic characteristics of offspring retained for analysis were statistically significantly different from those of offspring not retained, the differences were not substantively important with one exception (Table 1). In the father database, 49% of offspring retained for analysis had fathers who were university-educated, compared to 40% of those not retained.

**Table 1:** Comparison of selected socio demographic characteristics of offspring retained for analysis with those of offspring not retained, NDIT 2007-8

	**Mother database**			**Father database**		
	**Retained (n = 564)**	**Not retained (n = 316)**	**p-value for difference**	**Retained (n = 454)**	**Not retained (n = 426)**	**p-value for difference**
Age, y, mean (sd)	20.3 (0.7)	20.6 (0.8)	<0.0001	20.3 (0.7)	20.5 (0.8)	<0.0001
Male %	46.6	44.8	0.6604	47.0	44.7	0.5323
Mother university-educated, %	46.7	43.6	0.6654	49.2	39.5	0.0090
Household income, %						
<$30,000	36.7	47.5	0.0057	35.9	45.4	0.0002
$30,000-	44.7	41.5		42.9	44.5	
$99,999	18.6	11.0		21.3	10.1	
≥$100,000						
Canadian born, %	95.6	89.5	0.0009	95.8	90.8	0.0044
French-speaking,%	33.6	27.9	0.1774	28.4	33.6	0.1040

Mothers and fathers were aged 52 (sd=7) and 54 (sd=7) years on average, respectively at the time they completed the self-report parental questionnaires. Compared to fathers, higher proportions of mothers had been diagnosed with both mood and anxiety disorders, with the except of bipolar disorder (Table 2). Specifically, 23% of mothers had been diagnosed with either a mood or anxiety disorder, compared to 12% of fathers. Five percent of mothers had been diagnosed with both disorders, compared to 2% of fathers.

**Table 2:** Proportion of parents diagnosed with a mood and/or anxiety disorder, NDIT 2009-10

	**Mother (n=564) %**	**Father (n=454) %**
Mood disorder		
Depression	18.2	9.9
Bipolar	1.1	1.1
Postpartum depression	4.0	Not applicable
Any mood disorder	19.3	9.7
Anxiety disorder*	8.9	4.1
Mood and/or anxiety disorder		
Mood or anxiety disorder	23.0	11.8
Mood and anxiety disorder	4.8	1.6

*Includes phobias, obsessive compulsive disorder, panic attacks, and generalized anxiety disorder.

Similarly, statistically significantly higher proportions of female offspring (14%) had been diagnosed with either a mood or anxiety disorder, compared to male offspring (6%) (Table 3). Symptoms of social phobia were the most frequently endorsed anxiety subtype among both male and female offspring. With the exception of social phobia symptoms (for which there was no statistically significant sex difference), higher proportions of female than male offspring endorsed each anxiety subtype.

**Table 3:** Proportion of offspring diagnosed with mood and/or anxiety disorders, and proportion with symptoms of specific anxiety disorders, NDIT 2007–8

	**Total (n= 564) %**	**Female (n=302) %**	**Male (n=262) %**	***p*-value for difference by sex**
Diagnosed				
Mood disorder	6.9	9.6	3.8	0.0116
Anxiety disorder	7.6	10.3	4.6	0.0180
Mood or anxiety disorder	10.3	13.9	6.1	0.0039
Mood and anxiety disorder	4.3	6.0	2.3	0.0530
Anxiety symptoms				
Panic disorder	15.7	21.6	8.8	<0.0001
Generalized anxiety disorder (GAD)	9.4	13.7	4.6	0.0004
Social phobia	24.0	26.5	21.2	0.1684

A diagnosis of mood and/or anxiety disorders in mothers was associated with a 2- and 4-fold increase in the odds of offspring having a diagnosed mood and anxiety disorder, respectively (Table 4). Maternal history was also statistically significantly associated with an increased likelihood of social phobia symptoms in offspring.

**Table 4:** Adjusted odds ratios (OR) and 95% confidence intervals (CI) for the association between diagnosed parental mood and/or anxiety disorders and mental health outcomes among offspring, NDIT 2007-10

	**n**	**Offspring diagnosis**		**Anxiety symptoms in offspring**		
		**Mood disorderOR**_**adj**_**(95% CI)**	**Anxiety disorderOR**_**adj**_**(95% CI)**	**Social phobiaOR**_**adj**_**(95% CI)**	**Generalized anxiety disorder (GAD)OR**_**adj**_**(95% CI)**	**Panic disorderOR**_**adj**_**(95% CI)**
Mother diagnosed with mood and/or anxiety disorder	564*	2.2 (1.1,4.5)	4.0 (2.1,7.8)	2.2 (1.2,4.0)	1.8 (0.7,4.1)	1.7 (0.8,3.5)
*p*-value		0.0263	<0.0001	0.0131	0.1966	0.1497
Offspring sex		2.4 (1.1,5.3)	2.4 (1.2,5.0)	1.4 (1.0,2.2)	3.6 (1.9,7.4)	2.6 (1.6,4.5)
*p*-value		0.0249	0.0183	0.0752	0.0002	0.0003
Mother university-educated (yes, no)		0.5 (0.2,1.0)	0.50 (0.2,1.00)	1.3 (0.9,2.0)	2.0 (1.1,3.6)	0.7 (0.4,1.2)
*p*-value		0.0542	0.0583	0.1614	0.0233	0.2050
Father diagnosed with mood and/or disorder**	454*	0.9 (0.2,2.8)	**	0.5 (0.2,1.1)	0.8 (0.2,2.2)	**
*p*-value		0.8862		0.1263	0.7112	
Offspring sex		3.6 (1.4,11.1)		1.7 (1.1,2.7)	5.9 (2.6,15.9)	
*p*-value		0.0115		0.0236	0.0001	
Mother university-educated (yes, no)		0.4 (0.2,0.9)		1.1 (0.7,1.8)	1.9 (1.0,3.8)	
*p*-value		0.0429		0.6206	0.0622	

* n varies because of missing data.

**Model did not converge and is therefore not reported.

Paternal history was not statistically significantly associated with any of the mental health outcomes in offspring (Table 4). There were no statistically significant interaction between duration of time spent living with father (mean (sd) = 20 (4) years) and paternal mood and/or anxiety disorders.

Supplementary analyses (Additional file 1: Table S1) suggested that a depression diagnosis in mothers was associated with a 3-fold, 2-fold and 2-fold increase in the odds of offspring having diagnosed anxiety disorder, panic and GAD symptoms, respectively. Further, a diagnosis of anxiety disorder in mothers was associated with a 3-fold, 5-fold and 2-fold increase in the odds of offspring having diagnosed mood and anxiety disorders, and social phobia symptoms, respectively. Finally, a bipolar disorder diagnosis in fathers was associated with a 13-fold increase in the odds of offspring having diagnosed anxiety disorder. With respect to the temporal sequence of parental and offspring diagnoses and symptoms, maternal age of diagnosis of anxiety and mood disorders (39 (sd=9) and 39 (sd=10) years, respectively) preceded the maternal age at which offspring were diagnosed with mood and anxiety disorders (54 (sd=7) and 56 (sd=7) years, respectively) and when offspring first had social phobia symptoms (53 (sd=7) years), GAD symptoms (53 (sd=6) years), and panic symptoms (53 (sd=6) years).

Similarly for fathers, paternal age of diagnosis of anxiety and mood disorders (44 (sd=12) and 42 (sd=11) years, respectively) preceded paternal age when offspring were diagnosed with mood and anxiety disorders (58 (sd=6) and 60 (sd=6) years, respectively) and when offspring first had social phobia symptoms (55 (sd=7) years), GAD symptoms (57 (sd=7) years), and panic symptoms (55 (sd= 8) years).

## Discussion

Despite differences in methodologies, the main finding of this analysis - the strong and statistically significant association between maternal history and mood and anxiety disorders in offspring – is consistent with extant population-based studies [40-42,44,48]. The magnitude of the associations observed concords with that reported in previous population-based and clinical studies [25-33,35,36,38,39]. In addition, maternal history was related to symptoms of social phobia in offspring.

These findings emphasize the importance of ongoing research on familial clustering of mood and anxiety disorders. Despite investigation of conceptually credible mechanisms of the maternal history-offspring disorder association, which include genetic transmission [49-51], parenting behavior [52,53], and depressogenic child-rearing environments [54-57] recent reviews suggest that these mechanisms explain little of the variation in the association [23,52,53]. This has led to the studies that investigate parental history, parenting behavior, and familial environments concurrently [54,55]. More recent studies discuss other factors that may explain more of the variance in the maternal history-offspring disorder association including mindful parenting [58-60], positive maternal behaviours during problem-solving tasks with children, use of an avoidance conflict-resolution approach [55], positive offspring coping skills [61] and familial environments characterized by critical expressed emotion [62].

In this study, we did not detect any association between paternal history and offspring disorder, which supports several population-based studies [40,42], but not others [41,44]. This inconsistency may relate, at least in part, to methodological differences between studies. In contrast to several reports [40,42] , our study required that parents be diagnosed by a health care professional. Men seek treatment for mental health disorders much less frequently than women [63] and therefore some fathers in our sample could have met the diagnostic criteria for a mood and/or anxiety disorder, but never sought or received treatment. Further, paternal history may be related to offspring outcomes that were not within the scope of our study, such as conduct disorder and attention-deficit hyperactivity disorder [42,44]. Detection of an association between paternal and offspring disorders may have been limited by low power [19,64,65]. Finally, paternal and offspring mood and/or anxiety disorders may not be related, at least in our sample.

Our findings suggest that offspring with maternal mood and anxiety disorder histories should be monitored for social phobia symptoms, in addition to mood and anxiety disorders. Adequate screening and treatment of parents, and in particular, mothers, is also essential as parents are instrumental in effective treatment of children and adolescents with mood and anxiety symptoms and disorders [66]. Mothers can ensure that children do their homework, practice skills taught during treatment sessions, and monitor symptoms and response to interventions. Concomitant treatment of mothers with children being treated in clinics has improved outcomes in offspring [67].

Limitations of this analysis include that mood and anxiety diagnostic data were based on self-report rather than structured interviews. Misclassification of the disorders may have attenuated estimates of the association between parental history and offspring mental health disorders. Ascertainment of disorders using diagnosis by a mental health professional may not reflect all cases of disorder. However, we did not collect data on access to services or frequency of consultation. The use of what is, in essence, cross-sectional data limits causal inference. However, age of parental diagnoses preceded age of offspring diagnoses and onset of anxiety symptoms.

## Conclusion

In this study, maternal, but not paternal, mood and/or anxiety disorders were associated with mood and anxiety disorders in offspring. Depression among adults is a chronic, recurring disorder and therefore vigilance about its potential impact on offspring needs to be maintained. Clinically, it is critical when treating adults with depression to screen for symptoms in children, and similarly to screen for maternal disorders when treating children. Effective treatment of both offspring and parental disorders has been demonstrated, with optimal outcomes occurring when both are treated [68]. Future research should focus on ascertaining the precise timing and nature of the maternal symptoms that have the greatest negative impact on offspring. Further delineation of the natural course of depressive and anxiety disorders in high-risk offspring may help determine when and which symptoms first appear, which may lead to more timely intervention in both individuals and families.

## Competing interests

The authors declare no competing interests.

## Authors’ contributions

Contribution of authors: NL defined the study question, reviewed the literature, contributed to the design of the analysis and interpretation of data, and wrote sections of the article. ED, EC, DR, IK reviewed the literature, contributed to the design of the analysis and interpretation of data, and wrote sections of the article. DR contributed to data analysis and drafted the data analysis section in the article. JOL designed the study, obtained the funding, developed the survey instruments, supervised data collection, contributed to the design of the analysis and interpretation of data, coordinated drafting the article, and critically reviewed and edited sections of the article. All authors reviewed the article and approved the final version.

## Pre-publication history

The pre-publication history for this paper can be accessed here:

http://www.biomedcentral.com/1471-244X/12/188/prepub

## Supplementary Material

Additional file 1 **Table S1.** Adjusted odds ratios (OR) and 95% confidence intervals (CI) for the association between diagnosed parental mood and/or anxiety disorders and mental health outcomes among offspring, NDIT 2007–10. Click here for file
