# Supplementary material for: The association between parental history of diagnosed mood/anxiety disorders and psychiatric symptoms and disorders in young adult offspring
Source: BMC Psychiatry. 2012 Nov 5;12:188. doi: 10.1186/1471-244X-12-188 (PMC3534519; doi:10.1186/1471-244X-12-188)
Supplement: Additional file 1 — Table S1. Adjusted odds ratios (OR) and 95% confidence intervals (CI) for the association between diagnosed parental mood and/or anxiety disorders and mental health outcomes among offspring, NDIT 2007–10. [file 1471-244X-12-188-S1.docx]

**Table S1. Adjusted odds ratios (OR) and 95% confidence intervals (CI) for the association between diagnosed parental depression, bipolar and anxiety disorders and mental health outcomes among offspring, NDIT 2007-10**

|  | Offspring diagnosis | | Anxiety symptoms in offspring | | |
| --- | --- | --- | --- | --- | --- |
| Diagnosed parental disorder | Mood disorder | Anxiety disorder | Social phobia | Generalized anxiety disorder | Panic disorder |
|  | OR_adj*_ (95% CI) | OR_adj*_ (95% CI) | OR_adj*_ (95% CI) | OR_adj*_ (95% CI) | OR_adj*_ (95% CI) |
| Maternal depression | 2.0 (0.9-4.1) | 3.2 (1.6-6.3) | 1.2 (0.7-1.9) | 2.2 (1.1- 4.2) | 2.0 (1.1-3.4) |
| *p*-value | 0.0742 | 0.0007 | 0.4753 | 0.0149 | 0.0166 |
| Maternal bipolar | ** | ** | 1.7 (0.2-8.9) | 2.8 (0.1-20.8) | 1.8 (0.1-11.9) |
| *p*-value |  |  | 0.5524 | 0.3687 | 0.6046 |
| Maternal anxiety | 2.6 (1.0-6.0) | 5.3 (2.4-11.3) | 2.2 (1.2-4.0) | 1.8 (0.7-4.1) | 1.7 (0.8- 3.5) |
| *p*-value | 0.0384 | 0.0000 | 0.0131 | 0.1966 | 0.1497 |
|  | | | | | |
| Paternal depression | 1.3 (0.3- 4.1) | 1.3 (0.3- 4.2) | 0.6 (0.3- 1.4) | 1.1 (0.3- 3.1) | 1.5 (0.6- 3.5) |
| *p*-value | 0.6817 | 0.6647 | 0.2949 | 0.8396 | 0.3706 |
| Paternal bipolar | 4.3 (0.2- 32.3) | 13.5 (1.6 - 101.6) | 0.8 (0.0-5.4) | 2.3 (0.1- 19.6) | 4.7 (0.6- 31.0) |
| *p*-value | 0.2129 | 0.0101 | 0.8217 | 0.4885 | 0.1028 |
| Paternal anxiety | ** | 2.1 (0.3- 8.9) | 0.4 (0.1- 1.6) | ** | 2.1 (0.6-6.7) |
| *p*-value |  | 0.3529 | 0.2656 |  | 0.2133 |

*ORs adjusted for sex and mother university-educated (yes, no).

**Model did not converge and is therefore is not reported.
